# Carbolic Acid

**Published:** 1904-05

**Authors:** James F. Austin

**Affiliations:** St. Louis, Missouri.


### CARBOLIC ACID.

By James F. Austin, D.D.S., St. Louis, Missouri.

Read before the St. Louis Dental Society.

Acidum carbolicum (or carbolic acid) as we all know, plays a very important part in the treatment of some cases which present themselves to us. In discussing this powerful medicine, let us first notice what it is, what it is made from and how it is prepared. It is obtained from coal tar by fractional distillation, and is extracted from that portion of the heavy coal-tar oils which distil over between 200° and 400° F. When pure it appears in colorless crystals, and at 95° F. becomes an oily liquid, with a strong odor and taste. Chemically considered, carbolic acid is an alcohol rather than an acid. It was discovered by Runge in 1834 in the tar of coal. It is best kept for use in a dark amber bottle, tightly corked. Carbolic acid is useful in a great many ways in a dental office, and the desired results are obtained if properly used. In the first place, one cannot be too careful in the use of this powerful remedy. When treating the teeth the rubber dam should always be applied, in order to keep the acid from getting on the patient's face and scarring it. If, however, for any reason the dam is not applied and the acid should get on the face of the patient, immediately take some cotton and saturate it with absolute alcohol and apply it to the burn. In a minute or two the skin, if turned white, will become the natural color, and no bad effects will result. Carbolic acid, being an antiseptic, styptic, eschoratic, stimulant and sedative, is a very valuable agent to the dentist. It is used in my practice for obtunding sensitive dentin when preparing a cavity for filling. It will relieve severe toothache nine times out of ten almost instantly. When applied to an exposed pulp it arrests putrefaction and induces healthy action without irritation. I also use carbolic acid as a styptic in cases of superfacial hemorrhage from the gum when setting crowns and bridges—especially in setting Logan crowns, when there is always some hemorrhage if the root is ground as shor as it should be. In case of soft and spongy gums, carbolic acid diluted with alcohol will harden and make the gums less tender. There are two kinds of carbolic acid, the pure and the impure; and in treating putrescent anterior teeth, care should be used to get the chemically pure acid because if the impure (which has a reddish color) is used, a discolored tooth will be the result in a very short time. I find that Mallinckrodt's chemically pure acid is the best.

In the treatment of abscesses with carbolic acid, too much care cannot be exercised. In treating an abscess with a fistula, it is not necessary to run a stream of carbolic acid through it. A small quantity will accomplish just as good results. This I know by experience, for it has been my misfortune to have had a very thrilling episode to occur in using carbolic acid.

A patient presented herself to me for the treatment of an abscessed tooth with a fistula. The tooth had been crowned and the root broken off under the gum margin, and she had lost the crown. She said to me: "Now, Doctor, I want you to save this tooth and put another crown on it, if it can be done." I told her I thought I could do it. Taking the root-filling out of the canal, I took a small broach, and it would seemingly pass through the foramen at the apex of the root. The root being broken off under the gum margin, it was impossible for me to apply the rubber dam, so I took the hypodermic syringe and pumped some warm water through without any trouble. I was still afraid to try carbolic acid, so I took some glycothmoline and pumped it through. The water and gylcothmoline both went through so nicely that I thought I would pump some carbolic acid through and close the canal immediately. But instead of the acid going through as the others did, it shot off in another direction, going up into the cheek and face, which resulted in an abscess of the face. I extracted the root in a couple of days, and found it had been perforated. I could plainly see then how it all happened. In pumping the water and glycothmoline through I was not afraid of its coming back through the root and getting into the mouth; but when I went to pump the acid through, I pushed the needle as far as it would go, so as to have it as tight as I could get it in the root canal, in order to prevent the acid from getting into the mouth. The needle, of course, went through the perforation and into the gums, and the acid did likewise. Gentlemen, I know you will not be surprised when I tell you I lost the patient. I treated her the best I knew how for a week or ten days, and the last time I saw her I thought she was nearly well. Her face was swollen but very little, and the feeling seemed to be coming back into her lip and face. But she never returned to me. She went to one of the so-called ethical dentists of this city, and instead of him asking me what had happened, or what I had injected into the gum, he began to treat her for something he knew absolutely nothing about. He took out two good molars, one good bicuspid and a cuspid, cut away some of the alveolar process, and in time nature healed the wound. The same result would have obtained had he not extracted a tooth, but only kept an opening through which he could wash it out thoroughly with peroxid of hydrogen once every day.

---

DISCUSSION.

Dr. Walter Bartlett: In the treatment of alveolar abscess, I hardly think it necessary to use a hypodermic syringe. All that is necessary is to get a small amount of carbolic acid in the putrescent canal and, using a piece of soft rubber to get pressure behind, filling the cavity with the rubber and forcing the rubber up into the canal. To guard against the escharotic effects of carbolic acid as it comes through the fistulous opening, first dry off the gum thoroughly, and then paint glycerine over the surface of the gum, so that the glycerine may immediately take up any overflowing acid. I paint not only the gums, but the entire buccal surface. I find carbolic acid one of the most important drugs I have in my cabinet. I am not much of a druggist and don't believe in having a large display of medicines in a dentist's office, and believe we can get along without a small drug store. I use carbolic acid a great deal in preparing root canals for filling, and I have yet to find a root canal with a devitalized base that gives me trouble. After taking out the pulp, if I have hemorrhage I pour it in the canal and it coagulates the blood. I do not even take the precaution to wipe out thoroughly. As long as I have the odor of the acid in the canal, I go on filling. I cannot remember having had any bad results. I never think of treating the canal after the pulp is removed. For abscesses with fistulous openings, for the checking of hemorrhage in fitting bands, and sometimes in cavities where the dentin is sensitive, I take a pledget of cotton, thrust it into the spirit lamp, let it ignite, and while hot, place it in the cavity and leave it there. I find that in a great many cases I can cut sensitive dentin very much better after such treatment.

Dr. Prinz: There is one point of particular interest, and this is the application of alcohol as an antidote to its use. It is one of the best antidotes we have for carbolic acid, and we should, therefore, keep in mind that in using carbolic acid with some other drug which contains alcohol in some form will neutralize its effect, viz.: the combination of carbolic acid and tincture of iodine is valueless as far as the carbolic acid is concerned. Another very useful antidote for internal administration is vinegar. The best medium for liquefying carbolic acid for dental purposes is glycerine. From this solution the various aqueous solutions can be readily prepared up to a six per cent. solution without difficulty. I have found and and advocated for some time past that a combination of carbolic acid with thymol and a small quantity of camphor is an extremely valuable mixture for the treatment of putrescent root canals. Thymol, which is also a phenol, like carbolic acid, but rather insoluble in water, is more lasting in its effects, while the addition of camphor merely modifies the caustic effect of the two other drugs. This mixture should not, however, be confounded with camphophenique, which is only a mixture of carbolic acid, camphor, alcohol and glycerine, and very weak as an antiseptic, although non-irritating.

---

Dr. Conrad: Carbolic acid is a drug that is used daily, almost hourly, and I consider it, thereofre, as one of the most important in our medicine chest. The essayist said that carbolic acid should have a distinctive odor. Pure carbolic acid has very little odor, and pure carbolic acid must be white. Nor is it necessary to keep it in a dark place nor in a dark bottle. It can be placed in the sunlight, and pure white carbolc acid will never discolor. The coloring matter is an impurity and should be removed by the chemist. For that reason I buy only a certain brand, which never discolors. One of the great objections to carbolic acid in the past was that it discolored teeth. That was due to the fact that we all used an acid that was impure. I use the acid to dry out cavities when preparing for a filling. Nothing does this as clean and nicely as carbolic acid. I use it also to ascertain the extent of decay; it acts as a magnifier and brings out all the defects in the teeth. I also use it in diseases of the interdental spaces. Place carbolic acid, full strength, on a pledge of cotton in those spaces and leave it in the position, and nothing will so rapidly induce the healing of the gums. Dr. Prinz said that a five per cent. aqueous solution of carbolic acid is a perfect solution. I do not think he can make a five per cent. or even a three per cent. solution of carbolic acid which will be free from globules of acid floating in the bottle. A two per cent. solution of carbolic acid is the highest solution that can be called a perfect solution. I use carbolic acid in the treatment of devitalized teeth, and also in putrescent canals, but I do not "pump" it into the canal. If it is used carefully in its full strength it will form a coagulum with the gangrenous matter, and the entire mass can be pulled out. After once applied, I never fear any other trouble. Campho-phenique is non-escharotic—not by reason of the addition of glycerine, but because the mixture is united by distillation. A dishonest druggist may sometimes sell you campho-phenique of his own make, and this is escharotic to the full extent, as carbolic acid is. It seems to me strange that anyone would take a hypodermic syringe and ram it into an opening and squirt the contents into it. It's a wonder the patient did not die. The way to treat these false openings is to introduce the carbolic acid into the sanal on cotton and force it through. I use soft gutta-percha for this purpose. After a treatment or two we can force the gutta-percha into the false opening without trouble.

---

Dr. Prinz. Dr. Conrad said that carbolic acid was soluble only to the extent of two per cent. in water. The United States Pharmacopia states that carbolic acid is soluble to the extent of one part in about fifteen parts of water, which would make approximately a six and a half per cent. solution. Further, as a matter of fact, even the purest carbolic acid is apt to become pinkish, or even brown, under the influence of light. This may be also due to iron, either coming from the containers or from the pliers dipped into it., It does not depreciate its value in any degree.

---

Dr. Austin: In regard to getting carbolic acid on the face, I do not hope that this shall occur, but I mention it so that if it should happen we will know that alcohol will remove the effects. Of course, I would not advocate the use of a hypodermic syringe for its application. In the case mentioned, I used the syringe because I could not get at the trouble in any other way, as the root was broken off under the gum margin.

---

American Alkalometry, Vol. III. A Digest of Clinic Teachings for 1900, 1901. Editors: Dr. W. C. Abbott, Dr. W. F. Waugh. The Clinic Publishing Co., Chicago.

This volume presents a condensed outline of two years of practice with the Alkaloids and active principles. Much good matter is presented in the volume which will be of interest to the dentist. Dr. E. L. Clifford has an article on Alveolar Abscess and its causes and treatment, which should be valuable to the medical practitioner.

---

Manual of Materia Medica and Pharmacy. Specially designed for the use of Practitioners and Medical, Pharmaceutical, Dental, and Veterinary Students. By E. Stanton Muir, Ph.G., V.M.D., Instructor in Comparative Materia Medica and Pharmacy in the University of Pennsylvania. Third edition, Revised and Enlarged. Crown Octavo, 192 pages, Interleaved throughout. Bound in extra cloth, $2.00 net. F. A. Davis Company, publishers, 1914–16 Cherry St., Philadelphia, Pa.

In this work the author gives in a concise and clear manner those points which are of value to the student and practitioner of medicine, without the lengthy detail usually found in text-books, and only those drugs and preparations are considered which are in everyday use. The drugs are arranged in alphabetical order, making it very handy for reference work.

It is divided into three parts, taking up General Considerations, Individual Drugs, and Pharmacy. It is a book which every young dentist should secure and study carefully.

